# ST-Elevation Myocardial Infarction in a Young Man With Moyamoya Disease

**DOI:** 10.1016/j.jaccas.2025.106287

**Published:** 2025-12-03

**Authors:** Mina Yamashita, Hideki Okayama, Tsukasa Kurokawa, Go Kawamura

**Affiliations:** Department of Cardiology, Ehime Prefectural Central Hospital, Ehime, Japan

**Keywords:** cardiovascular disease, imaging, myocardial infarction

## Abstract

**Background:**

Moyamoya disease causes vasculopathic changes not only in intracranial arteries but also in extracranial vessels, including coronary arteries.

**Case Summary:**

A 25-year-old man collapsed from ventricular fibrillation and was resuscitated. Coronary angiography revealed left anterior descending artery occlusion, and optical coherence tomography demonstrated diffuse intimal thickening and thrombus formation without plaque rupture. Percutaneous intervention restored coronary flow. Subsequent evaluations revealed vasospastic angina and moyamoya disease.

**Discussion:**

This case highlights the unique pathophysiological mechanism of acute myocardial infarction in moyamoya disease, where fibrous intimal thickening predisposes to coronary spasm and thrombus formation, distinct from traditional atherosclerotic processes. Optical coherence tomography played a key role in identifying plaque morphology, guiding diagnosis, and management.

**Take-Home Message:**

Recognition of systemic vascular disorders, such as moyamoya disease, is essential in young patients with acute coronary syndrome to improve outcomes and prevent recurrence.

## Visual Summary

**Figure:**
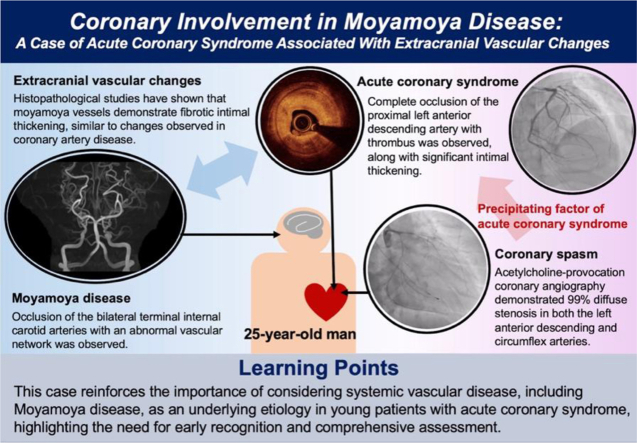

**Visual Summary:**
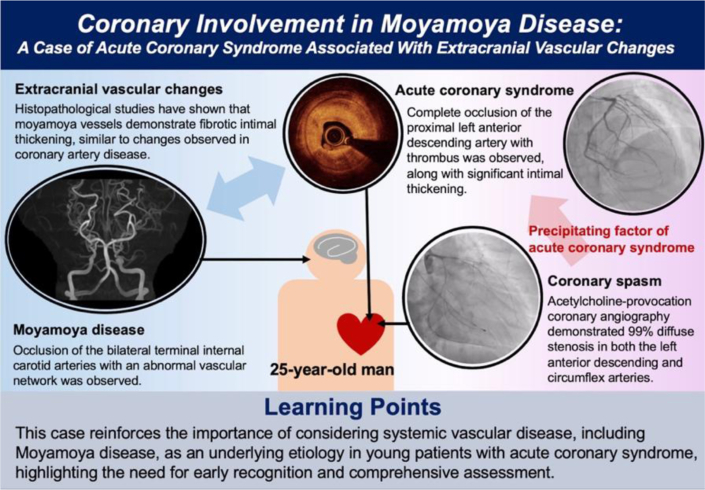
Coronary Involvement in Moyamoya Disease

## History of Presentation

The patient was a 25-year-old man who collapsed at his workplace, prompting an emergency call. Emergency medical services arrived to find him in ventricular fibrillation on electrocardiogram (ECG), and 2 defibrillations attempts were performed. Return of spontaneous circulation was achieved, and he was subsequently transferred to our hospital. On admission, he was intubated and unresponsive, with a blood pressure of 130/70 mm Hg, a heart rate of 124 beats/min in sinus rhythm, and an oxygen saturation of 97% on 60% inspired oxygen. Lung sounds were clear, no cardiac murmurs were audible, and there was no Achilles tendon thickening.

## Past Medical History

He was a former smoker without a history of hypertension, diabetes mellitus, or dyslipidemia. He reported occasional chest pain at rest, and with a clinical suspicion of coronary spastic angina, his primary physician initiated treatment with nifedipine.

## Differential Diagnosis

Sudden cardiac arrest due to ventricular fibrillation in a young man should prompt a differential diagnosis that includes acute myocardial infarction, coronary spastic angina, various forms of cardiomyopathy, and inherited arrhythmic disorders such as long QT syndrome and Brugada syndrome.

## Investigations

**Figure 1:**
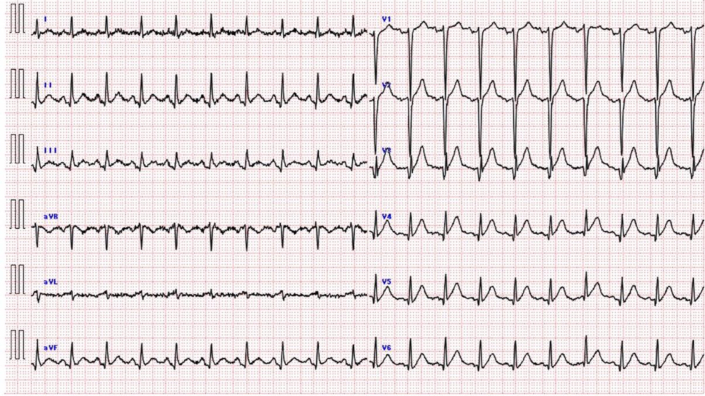
Electrocardiogram The first electrocardiogram in the emergency department showed sinus rhythm with ST-segment elevation across leads V_1_ to V_6_, with a preserved QTc interval.

An ECG obtained in the emergency department demonstrated ST-segment elevation in leads V_1_ to V_6_ with a normal QTc interval (Figure 1). Laboratory evaluation revealed a white blood cell count of 30,530/μL, high-sensitivity troponin I of 529 pg/mL, creatine kinase-myocardial band of 81 U/L, B-type natriuretic peptide of 7.9 pg/mL, D-dimer of 6.7 μg/mL, and low-density lipoprotein cholesterol of 120 mg/dL. Other laboratory tests and chest radiography were unremarkable. Transthoracic echocardiography showed a reduced left ventricular ejection fraction, with regional wall motion abnormalities extending from the anterior to lateral segments. No significant valvular abnormalities were detected, and myocardial wall thickness was within normal limits.

## Management (Medical/Interventions)

**Figure 2:**
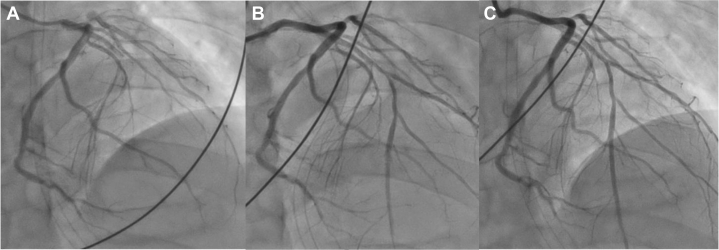
Coronary Angiography (A) Coronary angiography demonstrating total occlusion of the proximal left anterior descending artery (LAD). (B) Angiographic appearance of the LAD following thrombus aspiration. (C) Final angiographic result of the LAD after percutaneous coronary intervention with a drug-coated balloon.

**Figure 3:**
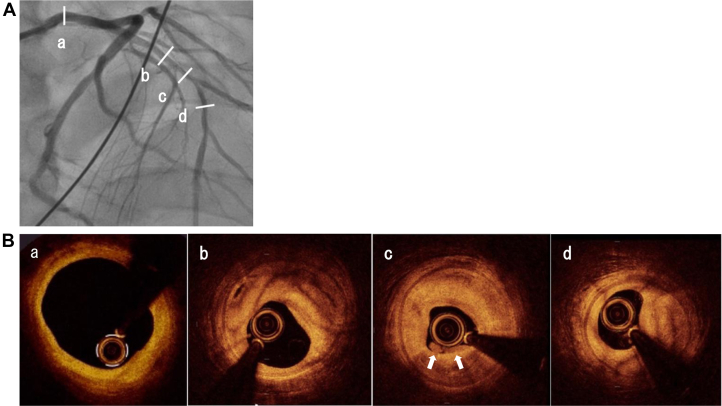
Optical Coherence Tomography Imaging (A) Coronary angiography at the time of optical coherence tomography (OCT). (B) OCT imaging. (a) OCT demonstrating no significant abnormalities in the left main coronary artery. (b-d) OCT at the culprit site showing marked intimal thickening due to fibrous plaque, layered plaque morphology, and white thrombus adherence (white arrows), without evidence of plaque rupture.

**Figure 4:**
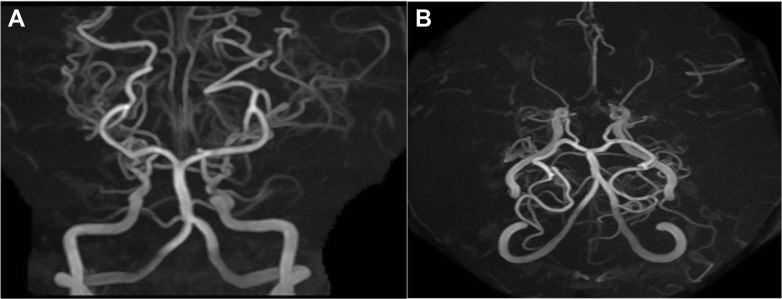
Head Magnetic Resonance Imaging (A and B) Head magnetic resonance imaging revealed occlusion of both terminal internal carotid arteries with associated abnormal collateral vascular networks, consistent with the diagnosis of moyamoya disease.

**Figure 5:**
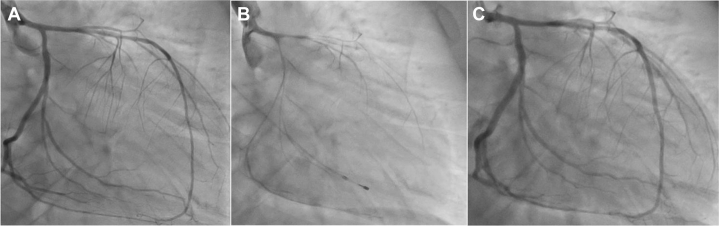
Acetylcholine-Provocation Coronary Angiography (A) Baseline coronary angiography. (B) Coronary angiography after acetylcholine injection demonstrating 99% diffuse vasospasm. (C) Coronary vasospasm resolved following nitroglycerin administration.

Given the high suspicion of ST-segment elevation myocardial infarction, emergency coronary angiography was performed, revealing a total occlusion of the proximal left anterior descending artery (LAD) with TIMI flow grade 0 (Figure 2). No additional significant lesions were identified. Following thrombus aspiration, coronary flow improved to TIMI flow grade 3, and optical coherence tomography (OCT) was performed to further evaluate the LAD. OCT demonstrated diffuse intimal thickening due to fibrous plaque, layered plaque morphology, and white thrombus adherence at the culprit site in the absence of plaque rupture, consistent with a definite intact fibrous cap (Figure 3). Scoring balloon angioplasty was successfully performed without recoil, followed by drug-coated balloon treatment. Targeted temperature management was maintained for 3 days, and the patient was extubated on hospital day 5. Benidipine was initiated in light of the patient's history and angiographic findings, raising suspicion for coronary spastic angina. Given the patient's young age, systemic vascular disease was considered. Brain magnetic resonance imaging revealed internal carotid artery stenosis with proliferative moyamoya vessels, and a diagnosis of moyamoya disease (MMD) was confirmed by neurosurgical evaluation (Figure 4). After rehabilitation, the patient was discharged on hospital day 21. Subsequently, acetylcholine-provocation coronary angiography induced 99% diffuse stenosis of the LAD and circumflex artery (Figure 5), accompanied by chest pain and corresponding ECG changes, establishing the diagnosis of coronary spastic angina.

## Outcome and Follow-Up

Medication has effectively controlled the patient's chest pain, enabling him to carry out daily activities without complications while continuing treatment under the supervision of his primary care physician.

## Discussion

MMD is a progressive cerebrovascular occlusive disorder of unknown etiology, characterized by stenosis or occlusion of the terminal portions of the bilateral internal carotid arteries and the development of abnormal collateral vascular networks. It is frequently associated with extracranial arterial diseases,^1^ and coronary artery lesions have been reported in 2.2% to 4.6% of patients with MMD.^2^^,^^3^ Histopathological studies have demonstrated fibrotic intimal thickening in moyamoya vessels, resembling changes observed in coronary artery disease.^4^ Although reports on coronary artery imaging in MMD are limited, intravascular ultrasound with virtual histology has confirmed the presence of fibrous plaques.^5^^,^^6^ In the present case, OCT provided detailed visualization of plaque morphology and facilitated speculation on the underlying mechanism of acute coronary syndrome. OCT demonstrated diffuse intimal thickening throughout the LAD, not confined to the culprit lesion, suggesting that the pathology may extend beyond coronary vasospasm to extracranial vascular involvement associated with MMD. Notably, no plaque rupture was identified; instead, a white thrombus was observed, consistent with an intact fibrous cap and OCT-defined erosion.^7^ The patient was subsequently diagnosed with vasospastic angina, and it was presumed that coronary vasospasm triggered plaque erosion, leading to the development of ST-elevation myocardial infarction.

## Conclusions

In young individuals, the presence of significant fibrous plaque lesions in the coronary arteries should prompt consideration of concomitant MMD.

## Funding Support and Author Disclosures

The authors have reported that they have no relationships relevant to the contents of this paper to disclose.

**Take-Home Message**

• This case reinforces the importance of considering systemic vascular disease as an underlying etiology in young patients with acute coronary syndrome, highlighting the need for early recognition and comprehensive assessment.

**Equipment List:** 

Coronary angiography	• Fluoroscopy unit (Philips Healthcare) • 0.035 J wire and 5-F slender sheath • 5-F JL3.5, 5-F JR4.0 (Terumo)
Percutaneous coronary intervention	• 6-F Mach1 coronary guiding catheter JL3.5 ST (Boston Scientific) • ASAHI SION blue PCI guidewire (Asahi Intecc) • ASAHI Caravel MC (Asahi Intecc) • 6-F Thrombuster PRO GR (Kaneka Medical Products) • MINAMO PCI guidewire (Asahi Intecc)
Imaging	• Dragonfly OpStar imaging catheter (Abbot Medical)
Balloon	• NSE advance PTCA balloon catheter 2.5∗13 mm (Goodman Co, Ltd) • SeQuentPlease NEO drug-eluting Balloon 2.5∗20 mm (Nipro) • SeQuentPlease NEO drug-eluting Balloon 2.75∗20 mm (Nipro)
